# Integrated Metabolomic and Transcriptomic Analyses of Anthocyanin Synthesis During Fruit Development in *Lycium ruthenicum* Murr.

**DOI:** 10.3390/biology14111614

**Published:** 2025-11-18

**Authors:** Jin Guo, Jing Wang, Chunxiang Peng, Hui Liu, Jie Shang

**Affiliations:** 1 College of Biological Science and Engineering, North Minzu University, Yinchuan 750021, China; guojinbf@163.com (J.G.); wangjing_imu@163.com (J.W.); 18395071022@163.com (C.P.); 20247657@stu.nmu.edu.cn (H.L.); 2 Innovation Team for Genetic Improvement of Economic Forests, North Minzu University, Yinchuan 750021, China

**Keywords:** anthocyanin, *L. ruthenicum*, metabolome, transcriptome, correlation network analysis

## Abstract

*Lycium ruthenicum* Murr. is a highly nutritional cash crop due to its fruit-abundant anthocyanins. With the development of the fruit, the color changes from green to dark purple and the anthocyanin content gradually increases. But the molecular mechanism of the anthocyanin biosynthesis process in *L. ruthenicum* fruit is still unclear. Five stages of *L. ruthenicum* fruit based on the color of the pericarp and flesh (BS1–BS5) were used for metabolomics and transcriptomics analyses to investigate the underlying mechanisms of the pigmentation. At the BS3 stage, the anthocyanin content was significantly increased and reached the highest level at the BS5 stage. A total of 25 DAMs related to flavonoids were identified by metabolomics, presenting a gradual increase with fruit development. Delphinidin-3-*O*-rutinoside and petunidin-3-*O*-rutinoside were identified as the main anthocyanins. Transcriptome sequencing and DEG analysis identified the key structural genes and transcription factors related to anthocyanin biosynthesis. Anthocyanin accumulation was driven mainly by the upregulation of six structural genes (*F3′5′H*, *DFR*, *ANS*, and *UFGT*) and eight key transcription factors from the HB, NAC, WRKY, Tify, AP2/ERF, and bHLH families that were significantly correlated with anthocyanin content in *L. ruthenicum* fruit. This study reveals key candidate genes in the anthocyanin biosynthetic pathway, providing new insights for improving fruit quality.

## Simple Summary

*Lycium ruthenicum* Murr. is a nutritious cash crop, valued for the abundant anthocyanins in the ripe fruit. With the development of the fruit, the anthocyanin content increases, but the molecular mechanism in this process is still unclear. This study investigated the process of fruit color change and anthocyanin accumulation at five developmental stages. Delphinidin-3-*O*-rutinoside and petunidin-3-*O*-rutinoside were the most abundant anthocyanins in *L. ruthenicum* fruit identified by metabolomic analysis. Through integrated metabolomic and transcriptomic analysis, six key structural genes (*F3*′*5*′*H*, *DFR*, *ANS*, and *UFGT*) and eight transcription factors (*HB*, *NAC*, *WRKY*, *Tify*, *AP2/ERF*, and *bHLH*) were speculated to play an important role in this accumulation. This research reveals the key candidate genes in the anthocyanin biosynthetic pathway, providing new insights for improving the fruit quality of *L. ruthenicum*.

## 1. Introduction

Anthocyanins are naturally occurring, water-soluble flavonoid pigments that serve as significant secondary metabolites in plants. They impart vibrant colors to attract pollinators and aid seed dispersal, protect against environmental stresses, and contribute to disease resistance [1,2,3,4]. Valued for their potent antioxidant and free-radical-scavenging properties, anthocyanins are widely used as a healthier alternative to industrial pigments in food coloring [5,6,7]. The value of these compounds is exemplified by *Lycium ruthenicum* Murr. (*L. ruthenicum*), an edible plant with berries rich in anthocyanins. The predominant monomer in *L. ruthenicum* fruit is petunidin-3-p-coumaroyl-rutinoside-5-glucoside (Pt5G) [8]. Studies of *L. ruthenicum* anthocyanins have demonstrated a variety of biological activities. They mitigate radiation-induced injury in mice by improving hematological parameters and reducing pro-apoptotic protein expression [9], significantly lower postprandial blood glucose levels in diabetic models [10,11,12], and ameliorate atherosclerosis by modulating the gut microbiota and regulating inflammatory and lipid metabolism pathways [13].

The biosynthesis of anthocyanins begins with the phenylpropanoid pathway, which generates the key precursor coumaroyl-CoA through a cascade of reactions catalyzed by the core enzymes *PAL*, *C4H*, and *4CL*. Building upon this foundation, the conserved flavonoid synthesis pathway located on the surface of the endoplasmic reticulum is activated [14]. The early stages of this pathway involve enzymes such as *CHS*, *CHI*, *F3H*, and *F3*′*H/F3*′*5*′*H*, which progressively construct the flavonoid skeleton [15]. Notably, *CHS* expression correlates positively with the anthocyanin content; for instance, the fading of *Brunfelsia acuminata* petals from deep purple to pure white coincides with decreased *CHS* expression [16]. Conversely, the relative expression levels of *F3*′*H* and *F3*′*5*′*H* competitively direct metabolic flux, determining the final flower color. For example, in *L. ruthenicum* fruit, an increased ratio of *LrF3*′*5*′*H* to *LrF3*′*H* transcript levels also shifts metabolic flux toward the delphinidin pathway [17].

The late stage is dominated by enzymes such as *DFR*, *ANS*, and *UFGT*. *DFR* converts dihydroflavonol into colorless anthocyanidin, and its deficient expression often results in an inability to synthesize anthocyanins, making it the structural gene most closely associated with anthocyanin deficiency in various plants [18]. *UFGT* stabilizes anthocyanins through glycosylation, enabling further modification by acyltransferases. Finally, glutathione S-transferases (GSTs) play a crucial role in anthocyanin transport and stable accumulation [19].

In addition to structural genes, transcription factors (TFs), including the MYB, bHLH, and WD40 proteins, also play important roles in anthocyanin biosynthesis. The expression of the R2R3-MYB and bHLH regulatory genes specifically promotes anthocyanin accumulation in most cases [20,21]. For instance, RcMYB114, RcbHLH, and RcWD40 regulate anthocyanin accumulation by forming the MBW complex in rose [22]. In pepper (*Capsicum annuum* L.), CaMYBA can activate the expression of *CaMYC* and form CaMYBA-CaMYC-CaTTG1 to promote the expression of structural genes to increase the anthocyanin content [23]. In addition to MYB, bHLH, and WD40, some other TFs function as critical regulators. WRKY can bind to W-box cis-elements in the promoter of structural genes involved in anthocyanin biosynthesis. In litchii (*Litchi chinensis* Sonn.) fruit, *LcWRKY17* activates genes in the phenylpropanoid pathway (*LcPAL3*, *Lc4CL*, and *LcANS*), thereby enhancing anthocyanin accumulation and mitigating post-harvest browning [24]. Similarly, in *Dioscorea composita*, *DcWRKY11* promotes proanthocyanidin biosynthesis through a direct interaction with the promoter of the *AtTT2* gene [25]. NAC TFs constitute another pivotal regulatory layer, often integrating environmental cues with metabolic pathways. In grape, *VvNAC17* enhances drought tolerance and concomitantly increases the anthocyanin content by activating *VvDREB1A* and *VvUFGT* [26]. In peach, a cooperative module involving *PpNAC22*, *PpNAC100*, and the MYB activator *PpMYB10.1* has been shown to modulate anthocyanin accumulation [27]. Furthermore, members of the AP2/ERF family have been implicated in flavonoid metabolism. A positive regulator, *LbAP2/ERF089* from wolfberry *(Lycium barbarum*), activates *LbLAR* and *LbDFR* to stimulate anthocyanin synthesis [28]. Conversely, the *Arabidopsis* AP2/ERF factor *AtABI4* represses anthocyanin biosynthesis via a light-dependent pathway that involves the transcription factor *HY5* [29]. Finally, the HB family of TFs also plays an indispensable role. In *Aquilegia oxysepala*, a protein complex containing HB_PHD directly governs the expression of genes required for dark red sepal formation [30]. In purple lettuce *(Lactuca sativa* L.), the expression of HB family members is induced by sucrose and low-nitrogen conditions, leading to a subsequent increase in anthocyanin levels [31].

The molecular mechanism related to anthocyanin biosynthesis in *L. ruthenicum* has been progressively elucidated. Initial transcriptomic analyses identified core components of the MBW complex, including the *MYB* TFs *LrAN2* and *LrMYB113*, the *bHLH* factors *LrJAF13* and *LrAN1b*, and the WD40 protein *LrAN11* [17]. Subsequent research revealed that phytohormone signaling, specifically a sharp increase in abscisic acid (ABA) signaling during fruit development, induces the expression of *LrMYB1*, which in turn activates structural genes to promote anthocyanin biosynthesis [32]. Recent functional studies have further refined this model. For instance, overexpression of the *bHLH* factor *LrAN1b* significantly enhances anthocyanin accumulation [33]. While the structural genes *LrCHS* and *LrF3*′*5*′*H* have been confirmed as positive regulators, the MYB family member *LrAN2.1* was identified as a negative regulator of this pathway [34]. Intriguingly, a paralogous gene, *LrAN2*, exhibits an opposing function; its ectopic overexpression in the *L. ruthenicum* callus markedly stimulates anthocyanin biosynthesis, underscoring the functional divergence within this gene family [35].

Although existing studies have initially revealed the pivotal role of the MBW complex-centered transcriptional regulatory network in anthocyanin biosynthesis in *L. ruthenicum*, this classical model does not fully capture the regulatory mechanism. Anthocyanin accumulation is a highly dynamic, complex process that is regulated at multiple levels and by various intricate factors. We aim to investigate the key period of anthocyanin biosynthesis and elucidate its overall molecular characteristics, discover novel regulatory factors beyond the known MBW complex, and construct a multi-dimensional regulatory pathway map connecting ‘genes to metabolites’.

In this study, the metabolic and transcriptomic profiles of *L. ruthenicum* fruits at five developmental stages were systematically analyzed. The anthocyanins and genes involved in the anthocyanin biosynthesis pathway were identified, and their expression patterns were analyzed. It is believed that these findings will provide candidate genes for investigating the molecular mechanisms underlying anthocyanin biosynthesis in *L. ruthenicum* and provide useful information for *L. ruthenicum* breeding.

## 2. Materials and Methods

### 2.1. Plant Material

The *L. ruthenicum* fruits were collected from the germplasm resource garden at the Ningxia Lycium Research Institute (38°38′52″ N, 106°8′55″ S) from seven-year-old *L. ruthenicum* plants. Based on the color of pericarp and flesh, samples from five distinct developmental stages were selected: BS1 (30 days after pollination, green fruit), BS2 (35 days after pollination, the color breaker stage of fruit), BS3 (38 days after pollination, complete discoloration), BS4 (40 days after pollination, the early expansion and deepening of pigmentation), and BS5 (45 days after pollination, complete maturity). All experimental materials were rapidly frozen in liquid nitrogen, transported to the laboratory, and stored at −80 °C.

### 2.2. Anthocyanin Content Measurement

After making a few minor adjustments to the ratio of the extraction solutions, the technique reported by Li et al. [32] was used to measure the anthocyanin content of *L. ruthenicum* fruits at five developmental stages.

For each experiment, approximately 0.5 g of fruit was used. Following the addition of 1 mL of a 1% HCl–methanol solution, extraction was conducted at 4 °C for 12 h. After extraction, chloroform and ddH_2_O were added to the mixture in a 1:1:1 ratio. The solution was then centrifuged at 4 °C and 13,400× *g* for 5 min. Subsequently, the absorbance of the supernatant was measured at 530 nm using a microplate reader (HBS-ScanZ Full-wavelength microplate reader, Detie Biotechnology Co., Ltd., Nanjing, China). The total anthocyanin content was calculated using the equation of the standard curve obtained with cyanidin-3-*O*-glusoside (SC8740, Solarbio, Beijing, China). All measurements were taken from three biological replicates for each treatment.

### 2.3. Detection of the Metabolome

Fruit samples were collected at five developmental stages, with three biological replicates for each stage, resulting in a total of fifteen samples for the metabolomic analysis using the UPLC-MS/MS platform. Fruit powder was obtained by crushing freeze-dried samples in a mixer mill (MM 400, Retsch, Haan, Germany). The powder (0.05 g) was weighed and dissolved in 1.2 mL of a 70% methanol extraction solution. The solution was vortexed every 30 min for 30 s and repeated for 6 cycles. After centrifugation (8064× *g*, 3 min), the supernatant was collected and filtered using a microporous membrane (0.22 μm pore size) for UPLC-MS/MS analysis.

This analysis was conducted on an ExionLC AD UHPLC system (Agilent SB-C18 column, Santa Clara, CA, USA) coupled with an Applied (Shanghai Aibo Caishi Analytical Instrument Trading Co., Ltd., Shanghai, China) Biosystems 6500 QTRAP triple quadrupole mass spectrometer. The mobile phases were 0.1% formic acid in water (A) and acetonitrile (B). The gradient elution program was set as follows: the content of B was increased linearly from 5% to 95% over 9 min, held for 1 min, then returned to 5% in 1.1 min, followed by a 2.9 min re-equilibration period. Chromatographic conditions were set at a flow rate of 0.35 mL/min, a column temperature of 40 °C, and an injection volume of 2 μL. For mass spectrometry, electrospray ionization (ESI) was used with a spray voltage of 4500 V, a source temperature of 500 °C, and a nebulizer gas pressure of 25 psi. Data were acquired in multiple reaction monitoring (MRM) mode. The declustering potential (DP) and collision energy (CE) for each metabolite were optimized.

### 2.4. Identification and Quantitative Analysis of Metabolites

For each period, the metabolites eluted from the UPLC system were monitored using scheduled MRM. The MRM signals were processed and analyzed using Analyst 1.6.3 software (Metware Biotechnology Co., Ltd., Wuhan, China). Metabolites were identified and quantified using a commercially available standard metabolites database (Metware Biotechnology Co., Ltd., Wuhan, China). Subsequently, the peak area for each identified compound was used for principal component analysis (PCA) and orthogonal partial least squares-discriminant analysis (OPLS-DA). For two-group comparisons, differentially abundant metabolites were determined based on a Variable Importance in Projection (VIP) score ≥ 1 and an absolute Log2 fold change (|Log2FC|) ≥ 1.0.

### 2.5. RNA Extraction and Sequencing

Three biological replicates of *L. ruthenicum* fruits at each of the five developmental stages were analyzed. Samples were ground to a fine powder in liquid nitrogen, then extracted with a Total RNA reagent kit (Tiangen Biotech Co., Ltd., Beijing, China), according to the manufacturer’s protocol. RNA quality and concentration were evaluated using electrophoresis on a 1.2% agarose gel and a micro-spectrophotometer (Nano-500, ALLsheng Instruments Co., Ltd., Hangzhou, China). A total of 1 μg of RNA per sample was used to generate sequencing libraries (NEBNext UltraTM RNA Libaray Prep Kit for Illumina, NEB, Ipswich, MA, USA), according to the protocol. The library fragments were purified using the AMPure XP system (Beckman Coulter, Beverly, Brea, CA, USA). After PCR of the cDNAs, PCR products were purified (AMPure XP system), and library quality was assessed on the Agilent Bioanalyzer 2100 system (Agilent Technologies, Inc., Santa Clara, CA, USA). All products were sequenced on the Illumina platform by Metware Biotechnology Co. (Wuhan, China).

### 2.6. RNA-Seq Data Analysis and Annotation

The raw data obtained from the sequencing platform were filtered using fastp (version 0.23.2) to obtain high-quality clean reads for assembly and analysis. Transcript assembly was performed on the clean reads using Trinity v2.13.2. Deduplicated transcripts were aligned with the KEGG, NR, Swiss-Prot, GO, COG/KOG, and Trembl databases using DIAMOND v2.0.9, while the amino acid sequences were aligned with the Pfam database using HMMER to obtain annotation information from the seven major databases. Differentially expressed genes (DEGs) were considered at |Log2 fold change| ≥ 1 and FDR < 0.05. The DEGs among the samples were identified with DESeq2 for subsequent analysis. GO and KEGG analyses were performed using the Metware Cloud, a free online platform for data analysis [36] (https://cloud.metware.cn).

### 2.7. Integrated Analysis of Metabolome and Transcriptome

To elucidate the relationship between gene expression and metabolite accumulation, a correlation analysis was performed on the DEGs and DAMs identified. Gene-metabolite pairs with |PCC| ≥ 0.9 and a *p*-value < 0.05 were considered significantly correlated and were selected to construct a correlation network. The network was visualized using Cytoscape (v3.9.1) software.

### 2.8. Transcription Factor Analysis

iTAK (v1.5) software [37] was utilized to predict plant TFs. Subsequently, the Pearson correlation coefficients between the expression of TFs and total anthocyanin content were analyzed. TFs with |PCC| ≥ 0.90 and a *p*-value < 0.05 were selected for further study.

### 2.9. Analysis of Gene Expression Using Quantitative Real Time-PCR (qRT-PCR)

The expression of ten structural genes in the anthocyanin biosynthesis pathway and ten TF genes identified among DEGs was evaluated using qRT-PCR. *LrH2B1* was used as the internal reference gene, and cDNAs from the *L. ruthenicum* fruits at different developmental stages were used as the templates. The *PerfectStart* Green qPCR SuperMix (2×) (Transgen Biotech Co., Ltd., Beijing, China) was also used. The primers were designed using the NCBI platform. Each experiment was repeated three times, and the relative expression level of each gene was calculated using the 2^−ΔΔCt^ method. The primers are listed in Table S1.

### 2.10. Statistical Analysis

Each experiment was repeated three times. Data are shown as mean ± standard deviation. Statistical analysis was performed using one-way ANOVA with Duncan’s test in SPSS Statistics 27.0 (SPSS Inc., Chicago, IL, USA), and *p*-values < 0.05 were considered significant.

## 3. Results

### 3.1. Anthocyanin Levels During L. ruthenicum Fruit Development

**Figure 1:**
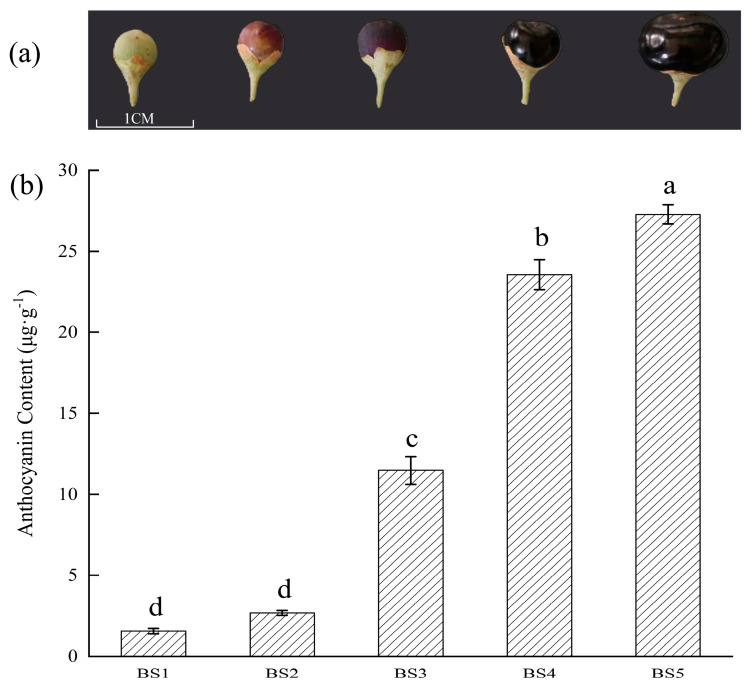
Diagrams showing the five developmental stages of *L. ruthenicum* fruit and the corresponding anthocyanin contents. (**a**) *L. ruthenicum* fruits at the five developmental stages. (**b**) Anthocyanin contents in *L. ruthenicum* fruit at the five developmental stages. The different letters above the bars indicate significantly different values (*p* < 0.05) calculated using a one-way analysis of variance (ANOVA) followed by Tukey’s multiple range test.

Obvious changes in both the physiology and morphology of the *L. ruthenicum* fruit were observed during its development. The color of the *L. ruthenicum* fruit skin changed from green to dark purple (Figure 1a). The anthocyanin content in the fruit changed significantly. The anthocyanin contents in *L. ruthenicum* fruit at the BS1, BS2, BS3, BS4, and BS5 stages were 1.56, 2.68, 11.48, 23.56, and 27.28 μg·g^−1^ (Figure 1b). A dramatic increase was observed from BS3 to BS4, suggesting that BS3 and BS4 represent the critical periods for anthocyanin biosynthesis and accumulation. This rapid synthesis phase is essential for the development of the fruit’s characteristic color.

### 3.2. Metabolite Analysis

The metabolomic analysis of *L. ruthenicum* fruit from the five developmental stages conducted using UPLC-MS/MS revealed significant changes in the metabolite profiles at each stage. Principal component analysis was performed using the samples from the *L. ruthenicum* fruits at the five developmental stages. The contribution rates of PC1 and PC2 were 48.41% and 12.43%, with a cumulative contribution rate of 60.81% (Figure S1a). The distribution of metabolites in the five developmental stages differed significantly, indicating clear differences in metabolites among the five developmental stages and the high reproducibility of metabolites among replicates.

A total of 1343 metabolites were identified and categorized into 11 types (Figure S1b): flavonoids (17.2%), alkaloids (16.53%), phenolic acids (15.12%), other metabolites (10.28%), lipids (9.46%), amino acids and their derivatives (8.64%), terpenoids (5.88%), organic acids (5.66%), nucleotides and their derivatives (4.62%), lignans and coumarins (4.24%), and steroids (2.38%). A heatmap was prepared using R (R-4.4.3) software (Figure S1c), and hierarchical clustering analysis was performed on the metabolite accumulation patterns. As shown in the figure, samples from the five developmental stages generally showed high repeatability within groups and consistent metabolite expression patterns. However, it is noteworthy that minor variability was still observed among biological replicates within the same developmental stage. This could be attributed to the intrinsic biological variation, as the same developmental stage is a relative concept. In practice, minor discrepancies in developmental timing among individual fruit may exist, which can lead to significant metabolic differences, especially during periods of rapid metabolic flux.

### 3.3. Analysis of the Differentially Accumulated Metabolites (DAMs) in L. ruthenicum Fruits at the Five Developmental Stages

A total of 812 DAMs were identified in *L. ruthenicum* fruits at the five development stages. A K-means clustering analysis was performed of all identified DAMs, categorizing them into five clusters (Figure S2a). Flavonoids and anthocyanins were primarily concentrated in Class 2 and Class 4 clusters, with their expression levels in Class 2 and Class 4 gradually increasing during fruit development. The patterns of metabolite synthesis in the two clusters were similar to the patterns observed in the changes in the overall anthocyanin contents of the fruit.

The DAM patterns in *L. ruthenicum* fruits from the four comparison groups (BS2 vs. BS1, BS3 vs. BS2, BS4 vs. BS3, and BS5 vs. BS4) were investigated. The numbers of DAMs in the pairwise comparisons were 134, 77, 278, and 238 in BS2 vs. BS1, BS3 vs. BS2, BS3 vs. BS4, and BS5 vs. BS4, respectively (Figure S2b). The numbers of metabolites in the BS4 vs. BS3 and BS5 vs. BS4 stages were significantly higher than those in the first two comparison groups. The BS3 and BS4 stages are key periods for fruit ripening, when the fruit color shifts from purple to deep purple, along with significant changes in metabolites, among which flavonoids are the most upregulated metabolites. In BS5, the fruit expands compared with BS4, and the most upregulated metabolites are alkaloids, which play a role in the response to biological stress [38].

**Figure 2:**
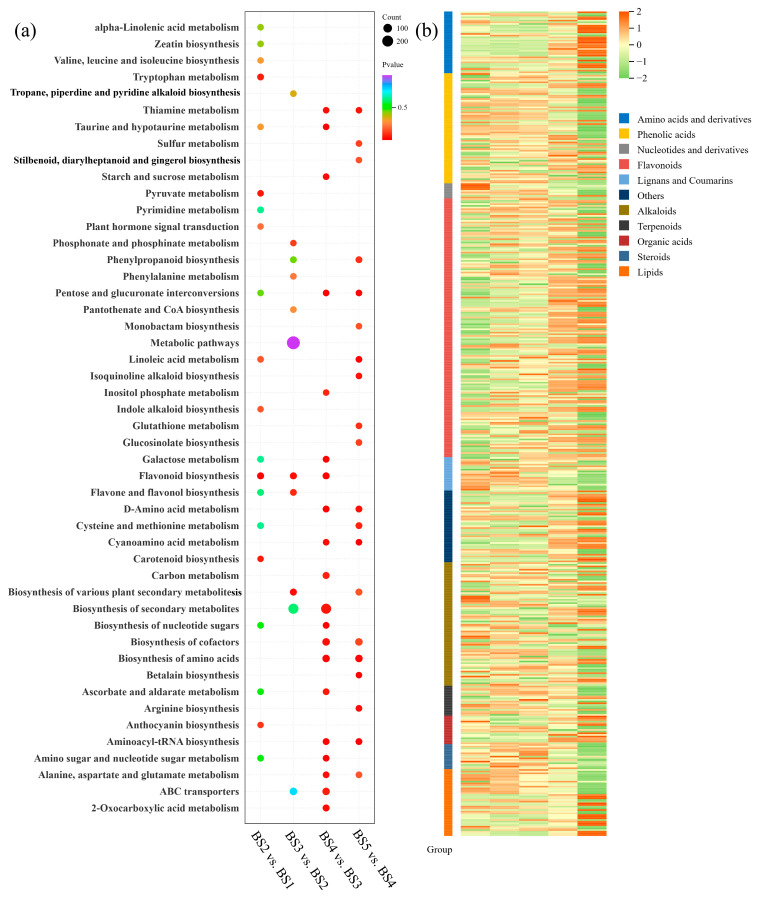

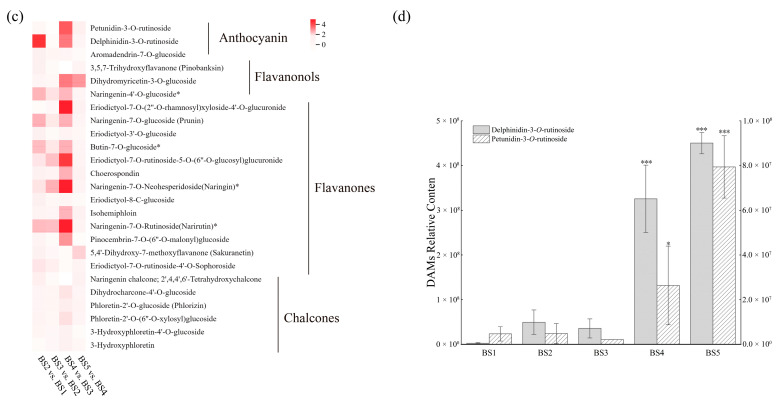
Metabolites showing differential accumulation during the development of *L. ruthenicum* fruit. (**a**) Top 20 significantly enriched KEGG pathways in DAMs from four comparison groups. (**b**) Variations in the relative levels of DAMs in the top 20 KEGG pathways. (**c**) Metabolites involved in anthocyanin synthesis. (**d**) The relative contents of anthocyanin metabolites. *, significant difference (*p* < 0.05); ***, highly significant difference (*p* < 0.001) (significance was determined by comparison with the BS1 group).

The significantly enriched metabolic pathways were identified by conducting a KEGG enrichment analysis of the DAMs. The top 20 enriched pathways of each comparison group are shown in Figure 2a. Specifically, in the BS2 vs. BS1 comparison, the top three significantly enriched pathways were flavonoid biosynthesis, pyruvate biosynthesis, and carotenoid biosynthesis. In the BS3 vs. BS2 comparison, the top three significantly enriched pathways were plant secondary metabolite biosynthesis, flavone and flavanol biosynthesis, and flavonoid biosynthesis. The top three significantly enriched pathways in the BS4 vs. BS3 comparison were D-amino acid metabolism, pentose and glucuronate interconversion, and flavonoid biosynthesis. These stages may provide more upstream metabolites for anthocyanin biosynthesis. The top three significantly enriched pathways in the BS5 vs. BS4 comparison were cyanoamino acid metabolism, aminoacyl-tRNA biosynthesis, and betalain biosynthesis. These findings closely align with the phenotypic changes observed in *L. ruthenicum* fruit. In the early growth stage (BS1–BS3), the fruit exhibits rapid growth owing to increased photosynthesis and energy metabolism, with the synthesis of flavonoids, flavones, and carotenoids aiding in stress resistance and inducing a color change. In the mid-growth stage (BS3–BS4), the synthesis of secondary metabolites and defense capabilities are increased in the fruit, with flavonoid and flavanol accumulation imparting a vibrant color to the fruit. In the mature stage (BS5), the metabolic activities shift to support fruit maturation. The metabolites derived from cyanoamino acid metabolism and betalain biosynthesis pathways may improve the fruit’s adaptability to environmental stress [39,40].

A heatmap of the relative DAM contents was prepared to further investigate the dynamic changes in the DAMs in the top 20 KEGG pathways identified for the four comparison groups (Figure 2b). The three most abundant types of DAMs were flavonoids, followed by alkaloids and phenolic acids, and their numbers were 155, 74, and 66. There were 96 flavonoids that were upregulated with fruit development, which were classified into seven categories: flavonols, flavones, flavanones, chalcones, flavanols, flavanonols, and anthocyanins. The relative flavonoid contents are shown in Table S2. Further screening of these flavonoids revealed that 25 metabolites were related to anthocyanin biosynthesis, and their expression patterns in the four comparison groups are shown in Figure 2c. Delphinidin-3-*O*-rutinoside and petunidin-3-*O*-rutinoside are two anthocyanins. Delphinidin-3-*O*-rutinoside was expressed in the BS1 stage first and its levels significantly increased in the BS4 stage, followed by the highest level in BS5 stage (Figure 2c). Petunidin-3-*O*-rutinoside accumulated in the BS1 stage and exhibited a notable increase in accumulation in the BS4 stage (Figure 2d). Delphinidin-3-*O*-rutinoside was the precursor of petunidin-3-*O*-rutinoside. There was a synergistic effect between the accumulation of delphinidin-3-*O*-rutinoside and petunidin-3-*O*-rutinoside.

### 3.4. Identification of DEGs in L. ruthenicum Fruit

**Figure 3:**
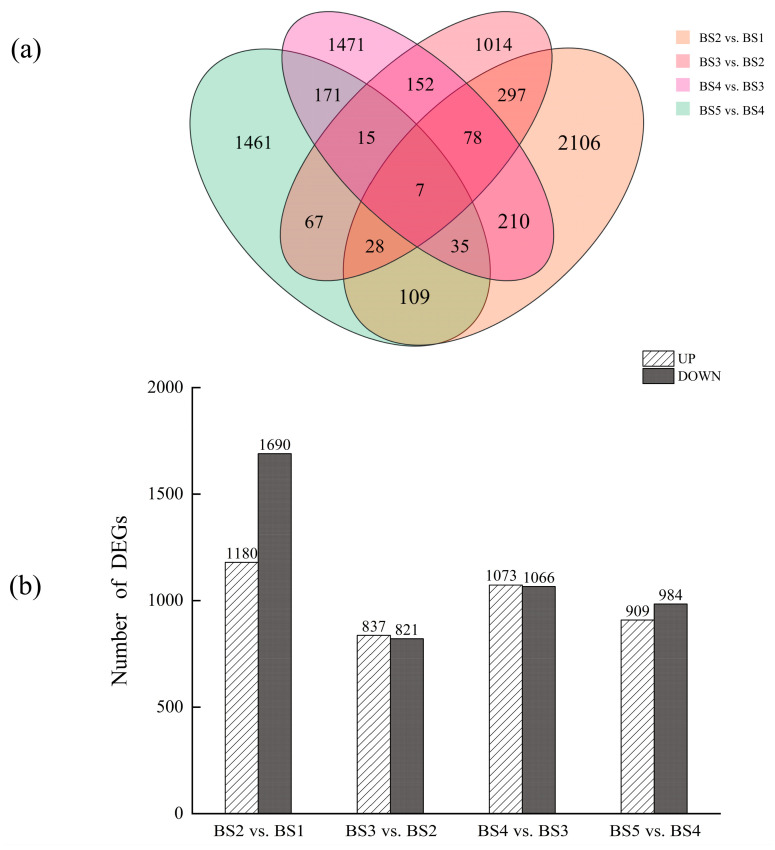
Variations in the expression of distinct genes during the development of *L. ruthenicum* fruit. (**a**) Venn diagram showing the DEGs between the four comparison groups. (**b**) Number of upregulated and downregulated DEGs between the four comparison groups.

After RNA-seq detection, 8560 DEGs (Figure 3a) in *L. ruthenicum* fruit from the four comparison groups were identified. The upregulated and downregulated DEGs are shown in Figure 3b. Based on the findings from the Venn diagram, 6052 genes that exhibited stage-specific expression were identified in the four comparison groups. These DEGs may play key roles during various periods of fruit development.

### 3.5. GO Analysis of DEGs

**Figure 4:**
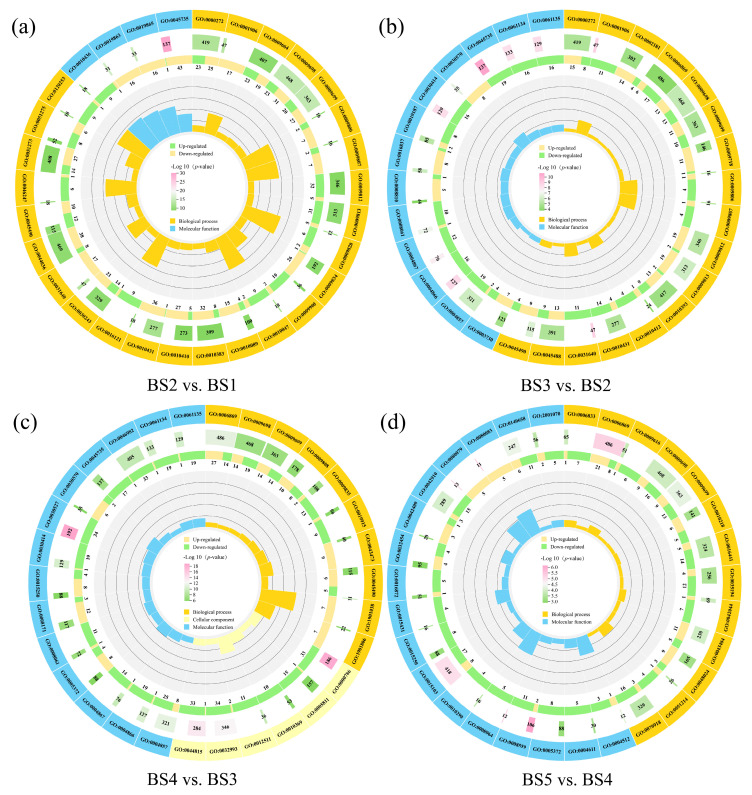
GO analysis of DEGs during the development of *L. ruthenicum* fruit. (**a**–**d**) GO enrichment results for the BS2 vs. BS1, BS3 vs. BS2, BS4 vs. BS3, and BS5 vs. BS4 comparisons. From the outer circle to the inner circle of the chart, the first ring represents the GO terms, with different colors representing different GO categories. The second ring shows the number of genes in the background within this classification and the Q-value. The longer the bar, the more genes are enriched, and the pinker it is, the more significant the enrichment. The third ring shows the proportions of upregulated and downregulated genes, with light yellow representing the proportion of upregulated genes and light green representing the proportion of downregulated genes; the specific values are shown below. The fourth ring shows the RichFactor values for each classification, with each small grid of the background auxiliary line representing 0.2.

The Gene Ontology (GO) analysis of DEGs during the development of *L. ruthenicum* fruit reveals a profound and dynamic evolution in their gene regulatory network across developmental stages. The 30 GO terms with lowest *p* values from each comparison group were analyzed here (Figure 4).

In the BS2 vs. BS1, BS3 vs. BS2, and BS5 vs. BS4 comparison groups, DEGs were enriched in biological processes and molecular functions, while in the BS4 vs. BS3 comparison group, DEGs were enriched in biological processes, molecular functions, and cellular components. Notably, DEGs were annotated in the phenylpropanoid biosynthetic process (GO:0009698) in four comparison groups, which means that this process was important in all stages of fruit development. In the BS2 vs. BS1 and BS3 vs. BS2 comparisons, DEGs were also annotated in the flavonoid biosynthesis process (GO:0009813), and the number of upregulated DEGs increased sharply, from 5 to 19. Flavonoid synthesis was active in BS3 vs. BS2 and may provide a large number of precursors for anthocyanin biosynthesis in the BS4 stage. In the BS4 vs. BS3 comparison, the cyanidin 3-*O*-glucoside metabolic process (GO:1901038) was enriched, and the seven DEGs were all upregulated, which means that anthocyanins were abundantly synthesized in this stage. DEGs enriched in these GO terms may be directly linked to anthocyanin biosynthesis, and the differences in the GO terms indicated the patterns of flavonoid and anthocyanin biosynthesis during the development of *L. ruthenicum* fruit.

The continuous increase in the number of enriched GO molecular function terms, namely, 4, 13, 14, and 17 in the four comparison groups, reflects the growing complexity of the regulatory demands for enzyme activity throughout development. In the BS4 vs. BS3 and BS5 vs. BS4 comparisons, DEGs enriched in *O*-methyltransferase activity (GO:0008171) and ABC-type glutathione S-conjugate transporter activity (GO:0015431) are perhaps related to anthocyanin biosynthesis.

### 3.6. KEGG Analysis of DEGs

**Figure 5:**
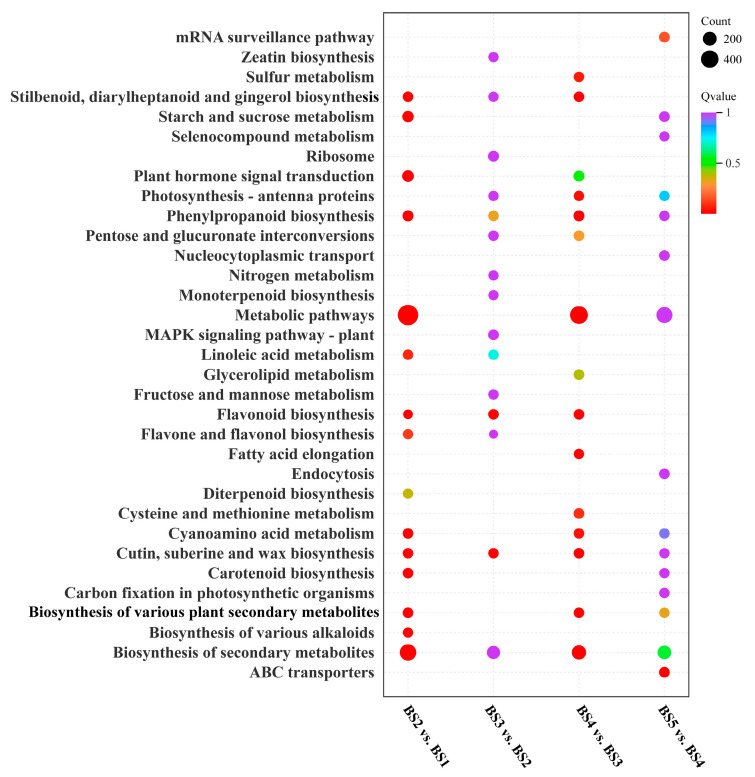
KEGG analysis of multiple combinations of DEGs.

KEGG pathway analysis was performed for the DEGs from the four comparison groups to conduct functional annotation and enrichment assessments (Figure 5). The degree of pathway enrichment was quantified by the Q-value and the number of enriched genes and was visualized in a bubble plot. An integrated analysis of the top 15 significantly enriched pathways for each comparison group revealed that *L. ruthenicum* fruit development is characterized by the intensive synthesis and accumulation of secondary metabolites. The enriched pathways were predominantly the biosynthesis of cutin, suberin, wax, flavonoids, phenylpropanoids, carotenoids, and alkaloids. These products collectively determine the fruit’s structure, color, flavor, and nutritional value, making them crucial for the formation of fruit quality. In particular, the flavonoid biosynthesis pathway was highly significantly enriched in three stages of fruit development (BS2 vs. BS1, BS3 vs. BS2, and BS4 vs. BS3). This finding clearly indicates that these specific developmental periods are critical windows for regulating flavonoid synthesis, during which numerous structural genes (e.g., PAL, CHS, and DFR) are synergistically activated, driving the massive accumulation of compounds such as anthocyanins. This stage-specific, high-intensity metabolic activity is the fundamental reason for the fruit’s characteristic deep purple color and exceptional antioxidant activity.

### 3.7. Integrated Analysis of the Metabolome and Transcriptome

**Figure 6:**
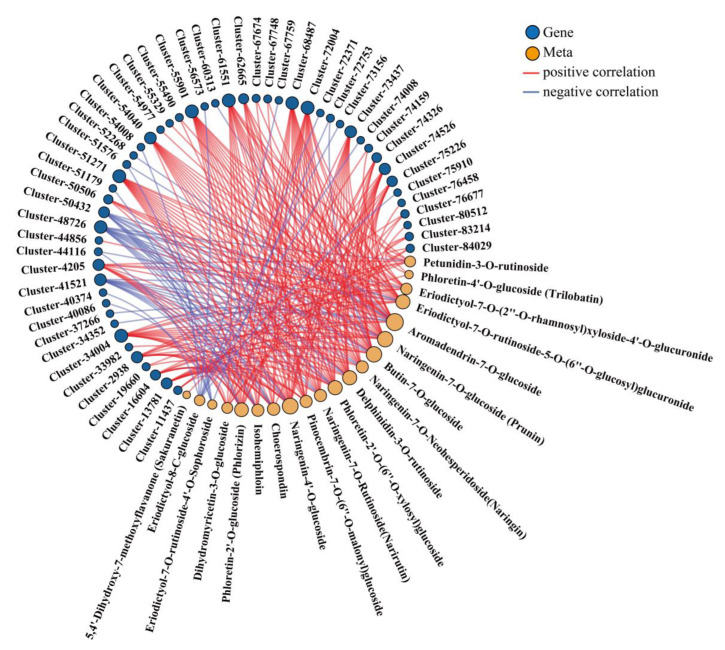
Network diagram of 25 flavonoid metabolites and their correlations with DEGs in the anthocyanin, flavonoid, and phenylpropanoid biosynthesis pathways.

A correlation analysis was performed to examine the relationships between DEGs and DAMs. Based on the transcriptomic results, the importance of the anthocyanin biosynthesis pathway, flavonoid synthesis pathway, and phenylpropanoid synthesis pathway in fruit quality formation is shown. The correlation network map revealed the association between the DEGs involved in the above pathways and flavonoid metabolites (|PCC| ≥ 0.90 and a *p*-value < 0.05). Through this analysis, a total of 52 genes were identified as being significantly correlated with flavonoid metabolites, among which 19 were structural genes for the anthocyanin biosynthesis pathway (Figure 6).

### 3.8. Analysis of Pathways Related to Anthocyanin Biosynthesis

**Figure 7:**
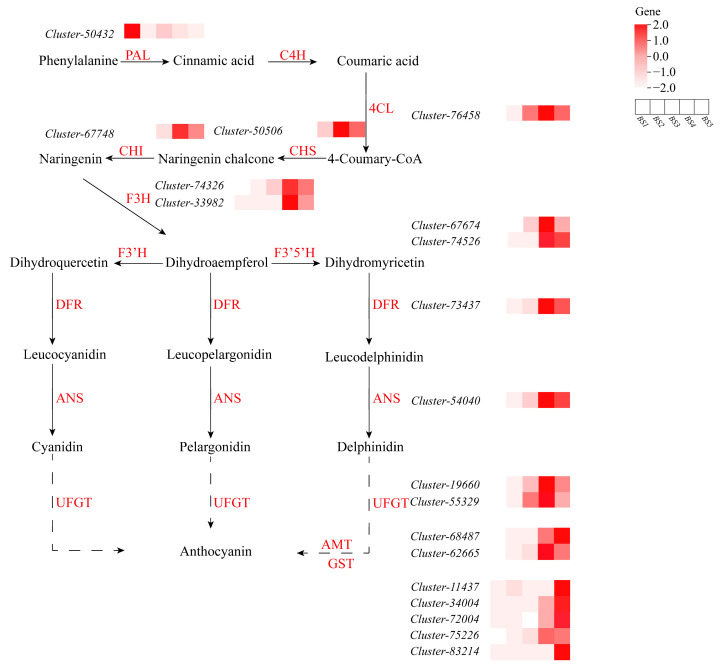
Anthocyanin biosynthetic pathway and expression levels of related genes in *L. ruthenicum* fruit.

A total of 19 differentially expressed genes (DEGs) associated with the anthocyanin biosynthesis and accumulation pathway were identified in *L. ruthenicum* fruit (Figure 7). These genes span the entire metabolic cascade, from the generation of precursors to the stabilization of the final pigment. The pathway begins with the phenylpropanoid stage, in which one *PAL* gene forms the backbone and one *4CL* gene activates intermediates for downstream synthesis. The core flavonoid pathway is initiated by one CHS protein, followed by one CHI protein and two F3H proteins that progressively construct the flavonoid skeleton. A critical branch point is governed by two F3′5′H proteins, which direct metabolic flux towards the synthesis of delphinidin pigments. The anthocyanin-specific pathway features one DFR protein, a crucial structural protein that reduces dihydroflavonols to leucoanthocyanidins, which is often indispensable for pigment formation. One ANS protein then oxidizes these leucoanthocyanidins to colored anthocyanidins. Finally, two UFGT proteins perform the essential glucosylation that stabilizes the anthocyanins, while two AMT proteins perform further modifications and five GST proteins facilitate transport and sequestration in vacuoles. As shown in Figure 6, the expression of the genes encoding these proteins was collectively and progressively upregulated during fruit development, with the most pronounced increases occurring in the late stages (BS3–BS4). Notably, *F3*′*5*′*H*, DFR, ANS, and *UFGT* exhibited the strongest upregulation at BS4 stage, corresponding with the phenotype of the fruit, identifying them as the genetic factors that drive the substantial accumulation of anthocyanins in *L. ruthenicum* fruit.

### 3.9. TFs Related to Anthocyanin Biosynthesis

TFs are important factors regulating anthocyanin accumulation. Through transcriptomic annotation, a total of 358 differentially expressed TFs were identified. Pearson’s correlation analysis identified 58 TFs associated with the total anthocyanin content in *L. ruthenicum* fruit based on |PCC| ≥ 0.90 and a *p*-value < 0.05, including 37 positively correlated TFs and 19 negatively correlated TFs. These TFs belonged to the MYB, NAC, WRKY, bHLH, AP2/ERF, HSF, LOB, TIFY, bZIP, and C2H2 families and may participate in the regulation of anthocyanin biosynthesis in *L. ruthenicum* fruit (Table S3).

**Figure 8:**
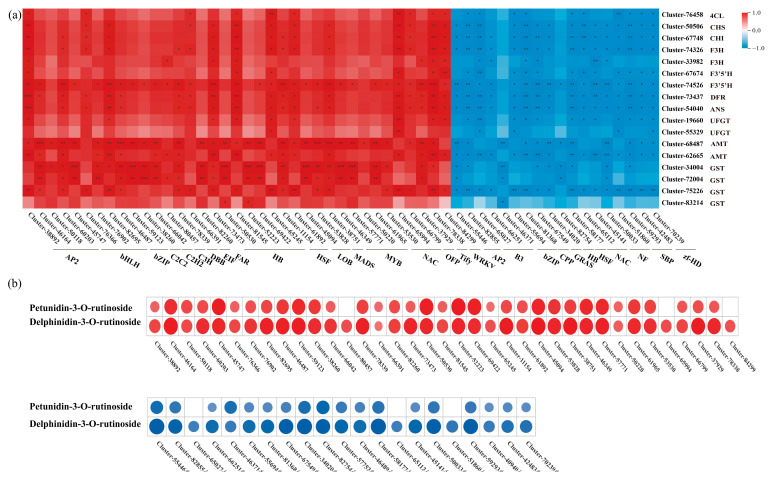
The correlations between anthocyanin metabolites, structural genes, and TFs. (**a**) Correlations between structural genes and TFs. (**b**) Correlations between anthocyanin metabolites and TFs, *, Significant difference (*p* < 0.05); **, Highly significant difference (*p* < 0.01); ***, Highly significant difference (*p* < 0.001).

To elucidate the transcriptional regulatory network of anthocyanin biosynthesis in *L. ruthenicum* fruit, a Pearson correlation analysis was performed between the expression profiles of 58 TFs and 26 structural genes (|PCC| ≥ 0.90, *p* < 0.05) (Figure 8a). This analysis identified 37 and 21 TFs that were positively and negatively correlated, respectively, with the structural genes, suggesting their potential roles as regulators in this pathway. Notably, eight TFs displayed strong positive correlations with most of the structural genes, including three members of the HB family (Cluster-73473, Cluster-81545, and Cluster-65245) and one each from the NAC (Cluster-65994), WRKY (Cluster-84299), Tify (Cluster-78338), AP2/ERF (Cluster-38892), and bHLH (Cluster-82695) families. Conversely, 11 TFs showed significant negative correlations with key structural genes such as *CHS*, *CHI*, F3H, *F3*′*5*′*H*, *DFR*, *ANS*, and *AMT*.

The eight TFs that showed strong positive correlations with structural genes emerged as key regulators of this pathway, demonstrating significant correlations not only with the structural genes but also with the total anthocyanin content (Table S3). To further validate this result, we analyzed their links to the metabolic phenotype and found that these TFs also showed significant positive correlations with the contents of major anthocyanins delphinidin-3-*O*-rutinoside and petunidin-3-*O*-rutinoside. Conversely, the negatively correlated TFs exhibited negative correlations with the contents of these metabolites (Figure 8b). These findings not only corroborate the regulatory role of these TFs in the biosynthesis pathway but also reveal a coherent regulatory cascade from the core transcriptional module through structural genes to the final accumulation of anthocyanin metabolites. Therefore, we speculate that the eight positively correlated TFs act as key regulators of anthocyanin biosynthesis and accumulation, likely by modulating the expression of the structural genes in the pathway.

### 3.10. qRT-PCR Analysis of Gene Expression

To validate the RNA-seq findings, qRT-PCR was performed on ten anthocyanin-related structural genes and ten TFs that showed strong correlations with the contents of anthocyanin metabolites (Figure S3). A comparison of the expression levels revealed strong consistency between the qRT-PCR and RNA-seq data for all selected genes.

## 4. Discussion

As the *L. ruthenicum* fruit develops, its appearance changes significantly, with its color changing from green to deep purple (Figure 1a). This process is closely associated with the gradual accumulation of anthocyanins during fruit development (Figure 1b). Previous studies have indicated that the anthocyanins in *L. ruthenicum* primarily include delphinidin derivatives, petunidin derivatives, and malvidin derivatives. Petunidin anthocyanins are the most abundant special anthocyanins, accounting for more than 78% of the total anthocyanin content [8]. The metabolomic analysis indicated that the types and levels of flavonoids increased gradually during fruit maturation, signifying the complexity and dynamics of fruit metabolic activities. In our study, two anthocyanins were detected. High delphinidin-3-*O*-rutoside and petunidin-3-*O*-rutoside contents were observed beginning at the BS3 stage and they peaked at the BS5 stage. Delphinidin-3-*O*-rutoside is the precursor of petunidin-3-*O*-rutoside. These findings are consistent with previous data from the fresh *L. ruthenicum* fruit [33,41]. In a previous study, petunidin-3-p-coumaroyl-rutinoside-5-glucoside (Pt5G) was the major anthocyanin component detected in *L. ruthenicum*, and was acylated by 3-p-coumaroyl-rutinoside to delphinidin-3-*O*-rutoside and petunidin-3-*O*-rutoside [42]. Delphinidin-3-*O*-rutoside and petunidin-3-*O*-rutoside are important upstream anthocyanins of Pt5G and significantly influence the fruit’s color and nutritional value.

The anthocyanin content in the fruit was closely correlated with the expression of structural genes and TFs involved in the anthocyanin biosynthesis pathway. In previous studies, the expression of *PAL*, *C4H*, *CHS*, *F3H*, *F3*′*H*, *F3*′*5*′*H*, *DFR*, and *ANS* played an important role in anthocyanin biosynthesis during *L. ruthenicum* fruit development [43,44], but information on *UFGT* and *GST*, which were important for anthocyanin modification and transportation, was insufficient. In many plants, the functions of UFGTs in color formation have been identified, such as *UFGTs* in sunflowers (*Helianthus annuus* L.) [45], *MiUFGT3* in mango [46], and *AnUFGT1* in *Anthurium* ‘Alabama’ [47]. In our study, two *UFGTs* (Cluster-19660 and Cluster-55329) were identified. Their expression levels increased sharply during the BS4 stage of fruit development (Figure 7), corresponding to the observed fruit phenotype. We speculate that this gene may participate in anthocyanin accumulation. *GST* is known to play a crucial role in the transport and accumulation of anthocyanins [19]. Furthermore, *GSTs* are related to anthocyanin biosynthesis in vitro [48]. In Japanese *Gentiana*, for example, the *GST1* gene is associated with the anthocyanin content but shows no significant expression in petals during early floral development stages 1–4 [49]. This pattern highlights that the critical function of *GST* genes is timed with the onset of anthocyanin synthesis. Consistent with this finding, our study identified five *GST* genes that were highly expressed specifically during the color breaker stage of fruit (BS2), the early expansion and deepening of pigmentation (BS4), and complete maturity (BS5) of fruit development, which means that they are important for anthocyanin accumulation. In addition to these genes, cytochrome P450, a superfamily of monooxygenases distributed extensively in nature, plays a crucial role in the synthesis of secondary metabolites, such as anthocyanins, in several plants [50]. Two particular cytochrome P450 enzymes, *F3*′*H* and *F3*′*5*′*H*, control the hydroxylation patterns and regulate flavonoid flux to the cyanidin or delphinidin branch [51]. In *L. ruthenicum* fruit, the ratio of *F3*′*5*′*H*/*F3*′*H* transcription increased sharply at the S4 stage and may determine the phenotypic difference in anthocyanin biosynthesis in *L. ruthenicum* [17].

TFs are pivotal regulators orchestrating the spatiotemporal dynamics of anthocyanin biosynthesis. In our study, the transcriptome analysis of *L. ruthenicum* fruit identified several TF families involved in this process. Among them, a bHLH protein (Cluster-82695) and a TIFY family protein (Cluster-78338) emerged as the most compelling candidates as key positive regulators. This conclusion is strongly supported by their exceptionally strong positive correlation with the anthocyanin content (r = 0.999 and 0.983) and their expression profiles, which precisely mirrored anthocyanin accumulation across all fruit developmental stages. Their minimal expression in early stages, followed by a dramatic surge coinciding with the anthocyanin peak, suggests they are principal drivers of anthocyanin biosynthesis during fruit ripening.

The identification of a bHLH TF as a top candidate aligns with the well-established MBW (MYB-bHLH-WD40) complex, the core regulatory module responsible for anthocyanin biosynthesis in most plants [52]. Within this complex, R2R3-MYB TFs typically confer specificity, while the bHLH partner is indispensable for complex stability and transcriptional activation [15,53]. For instance, in potato (*Solanum tuberosum*), *StbHLH1* forms a functional complex with *StMYB210* to regulate anthocyanin production [54]. The near-perfect correlation of Cluster-82695 with anthocyanin levels in *L. ruthenicum* suggests a similarly crucial role. We therefore speculate that this bHLH protein forms a regulatory complex with the previously identified *MYB*, *LrAN2* (Cluster-57771) [43,55], to co-activate structural genes in the anthocyanin pathway.

More intriguingly, the TIFY gene (Cluster-78338) exhibited an expression pattern nearly identical to the bHLH TF and a very strong correlation with the anthocyanin content. TIFY proteins, particularly JAZ subfamily members, are canonical repressors in the jasmonic acid (JA) signaling pathway [56]. In this model, JA accumulation promotes the degradation of *JAZ* repressors, thereby releasing MYC-type bHLH TFs to activate downstream genes. The strong positive correlation of Cluster-78338 with anthocyanin accumulation, in contrast to its typical repressive role, suggests a more complex regulatory function. We propose two non-mutually exclusive hypotheses. First, Cluster-78338 may represent a non-canonical TIFY protein that functions as a transcriptional activator, a role that has been observed for some *TIFYs* in other developmental contexts. Second, and more plausibly, its high expression may reflect a sophisticated feedback mechanism within the JA signaling circuitry. A surge in JA levels during ripening could trigger a massive transcriptional response of *JAZ* genes as part of the fine-tuning of this pathway, coinciding with the activation of anthocyanin biosynthesis. This tight co-regulation implies a sophisticated interplay between hormonal signaling and pigment production in *L. ruthenicum*, a mechanism that warrants detailed functional characterization.

## 5. Conclusions

This study elucidates the core regulatory mechanism of anthocyanin synthesis during fruit development in *L. ruthenicum* through integrated metabolomic and transcriptomic analyses. We identified the BS3 to BS4 stages as the critical periods for anthocyanin synthesis and accumulation, which correspond to the phenotypic change. During this window, the flavonoid biosynthesis pathway serves as the central metabolic conduit, driving the rapid accumulation of key anthocyanins like delphinidin-3-*O*-rutinoside and petunidin-3-*O*-rutinoside. Through a comprehensive correlation analysis, we pinpointed fourteen genes as key regulatory factors in this process: six structural genes, namely, *F3*′*5*′*H* (Cluster-67674 and Cluster-74526), *DFR* (Cluster-73437), *ANS* (Cluster-54040), and *UFGT* (Cluster-19660 and Cluster-55329), and eight TFs, namely, three HB family members (Cluster-73473, Cluster-81545, and Cluster-65245) and one each from NAC (Cluster-65994), WRKY (Cluster-84299), Tify (Cluster-78338), AP2/ERF (Cluster-38892), and bHLH (Cluster-82695). Collectively, these genes represent promising targets for fruit color improvement and provide an experimental basis for the breeding of elite *L. ruthenicum* varieties.

## Data Availability

The original contributions presented in this study are included in the article. Further inquiries can be directed to the corresponding author.

## Supplementary Materials

The following supporting information can be downloaded at: https://www.mdpi.com/article/10.3390/biology14111614/s1. Figure S1. Principal component analysis, classification of metabolites, and heatmap of relative content of all metabolites. Figure S2. Metabolite clusters and changes in the differentially accumulated metabolites (DAMs) during the development of *L. ruthenicum* fruit. Figure S3: Gene expression assay of qRT-PCR. Table S1: Primer list. Table S2: Relative content of flavonoids in *L. ruthenicum* fruit. Table S3: Expression profiles of transcription factors correlated with total anthocyanin.
