# Supplementary material for: Integrated Metabolomic and Transcriptomic Analyses of Anthocyanin Synthesis During Fruit Development in Lycium ruthenicum Murr
Source: Biology (Basel). 2025 Nov 18;14(11):1614. doi: 10.3390/biology14111614 (PMC12650669; doi:10.3390/biology14111614)
Supplement: Supplementary file 1 [file biology-14-01614-s001.zip › Figure S1.pdf]

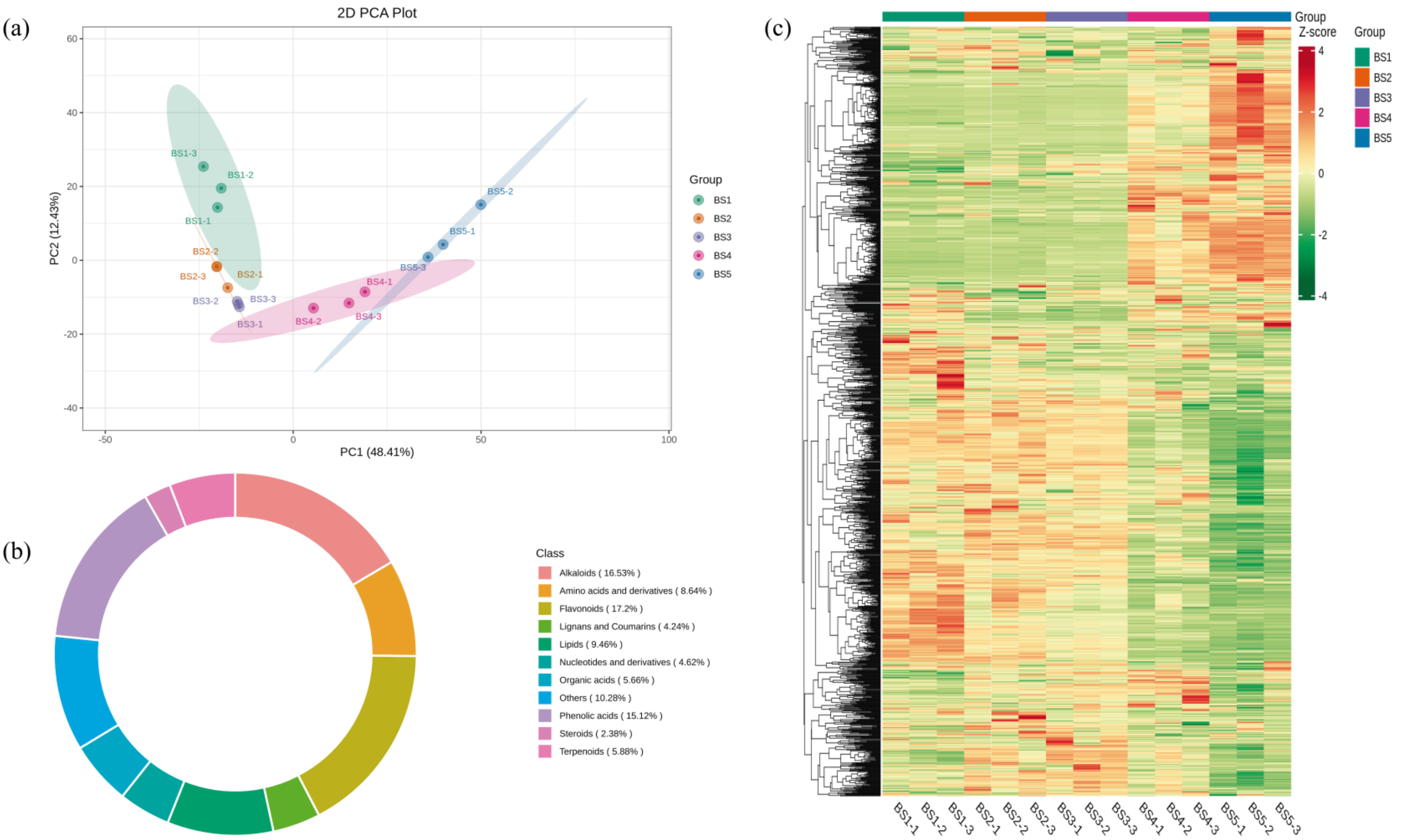

Figure S1. Principal component analysis, classification of metabolites and the heatmap of relative content of all metabolites. (a) Principal component analysis of metabolites. (b) Metabolites classification diagram. (c) The heatmap analysis of relative content of all metabolites in five different developmental stages of *L. ruthenicum* fruit.
