# Supplementary material for: Integrated Metabolomic and Transcriptomic Analyses of Anthocyanin Synthesis During Fruit Development in Lycium ruthenicum Murr
Source: Biology (Basel). 2025 Nov 18;14(11):1614. doi: 10.3390/biology14111614 (PMC12650669; doi:10.3390/biology14111614)
Supplement: Supplementary file 1 [file biology-14-01614-s001.zip › Figure S2.pdf]

(a)

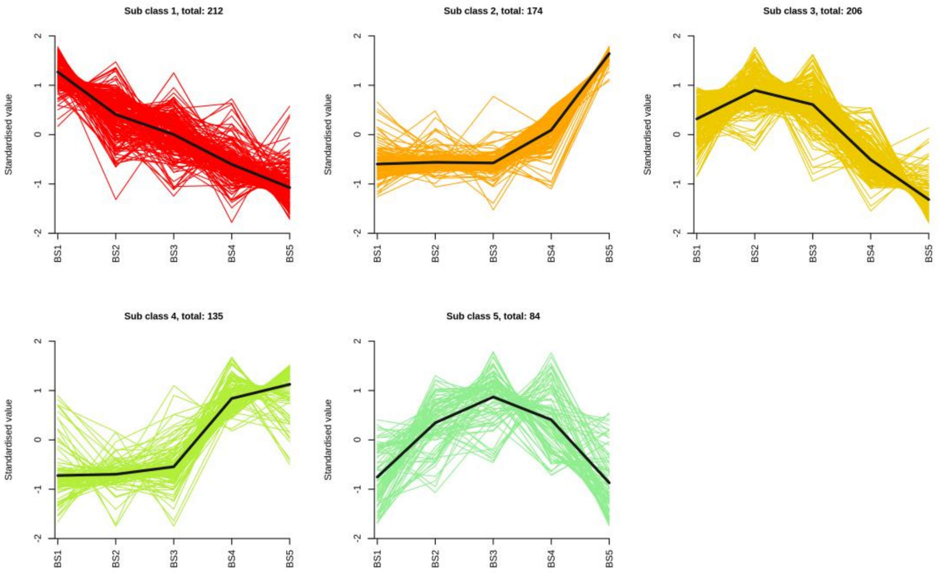

(b)

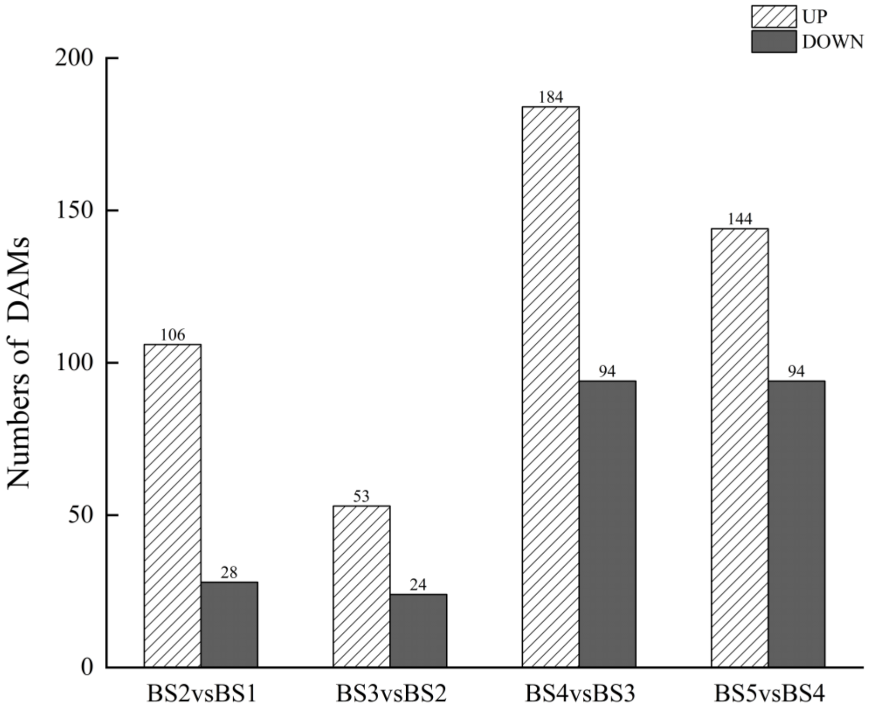

Figure S2. Metabolite clusters and changes of the differentially accumulate metabolites (DAMs) during the development of *L. ruthenicum* fruit. (a) K-means clustering analysis diagram of DAMs, (b) The number of DAMs at four comparison groups.
