# Supplementary material for: Integrated Metabolomic and Transcriptomic Analyses of Anthocyanin Synthesis During Fruit Development in Lycium ruthenicum Murr
Source: Biology (Basel). 2025 Nov 18;14(11):1614. doi: 10.3390/biology14111614 (PMC12650669; doi:10.3390/biology14111614)
Supplement: Supplementary file 1 [file biology-14-01614-s001.zip › Figure S3.pdf]

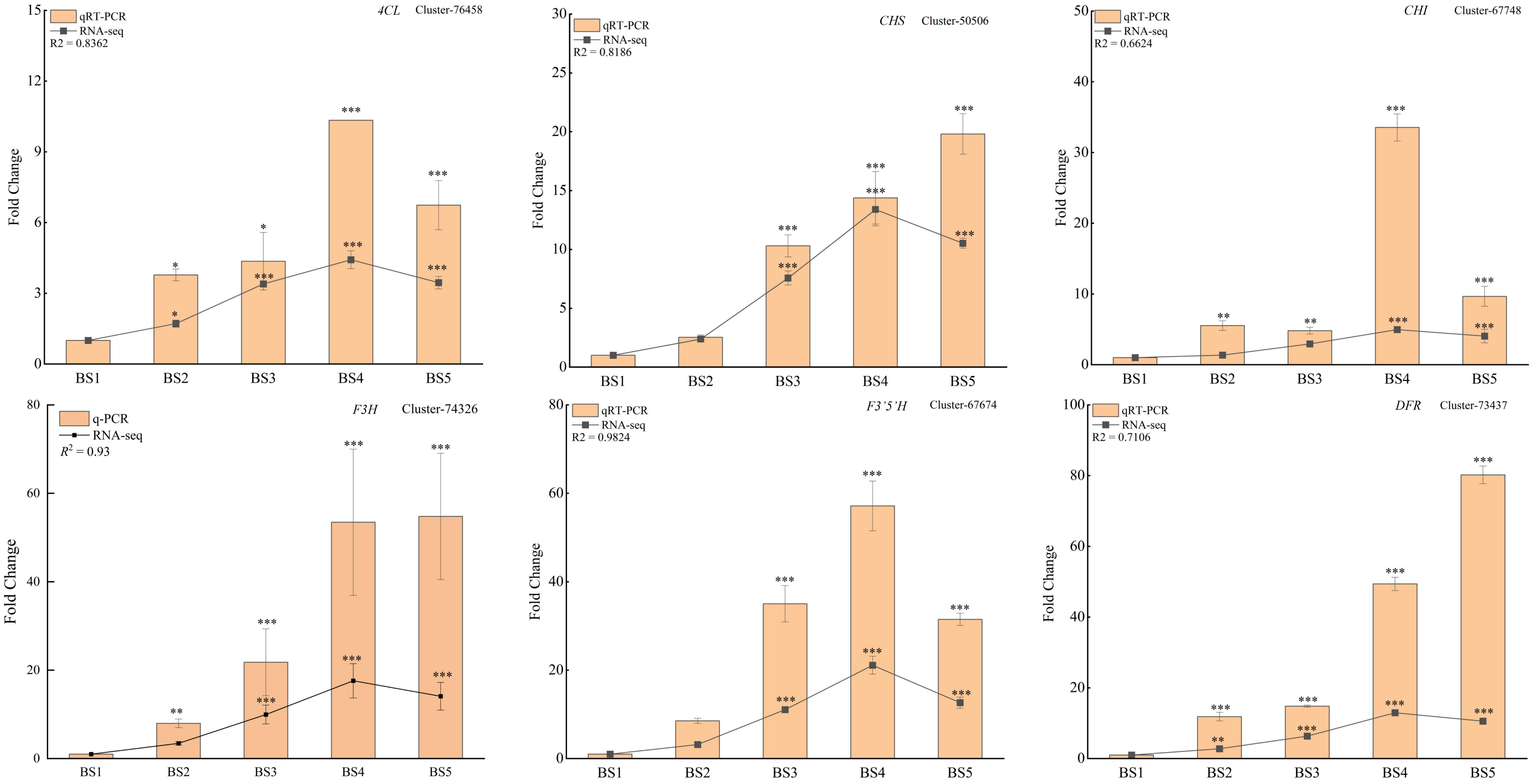
4CL Cluster-76458
qRT-PCR
RNA-seq
R2 = 0.8362
CHS Cluster-50506
qRT-PCR
RNA-seq
R2 = 0.8186
CHI Cluster-67748
qRT-PCR
RNA-seq
R2 = 0.6624
F3H Cluster-74326
q-PCR
RNA-seq
R^2 = 0.93
F3'5'H Cluster-67674
qRT-PCR
RNA-seq
R2 = 0.9824
DFR Cluster-73437
qRT-PCR
RNA-seq
R2 = 0.7106
Fold Change
BS1
BS2
BS3
BS4
BS5

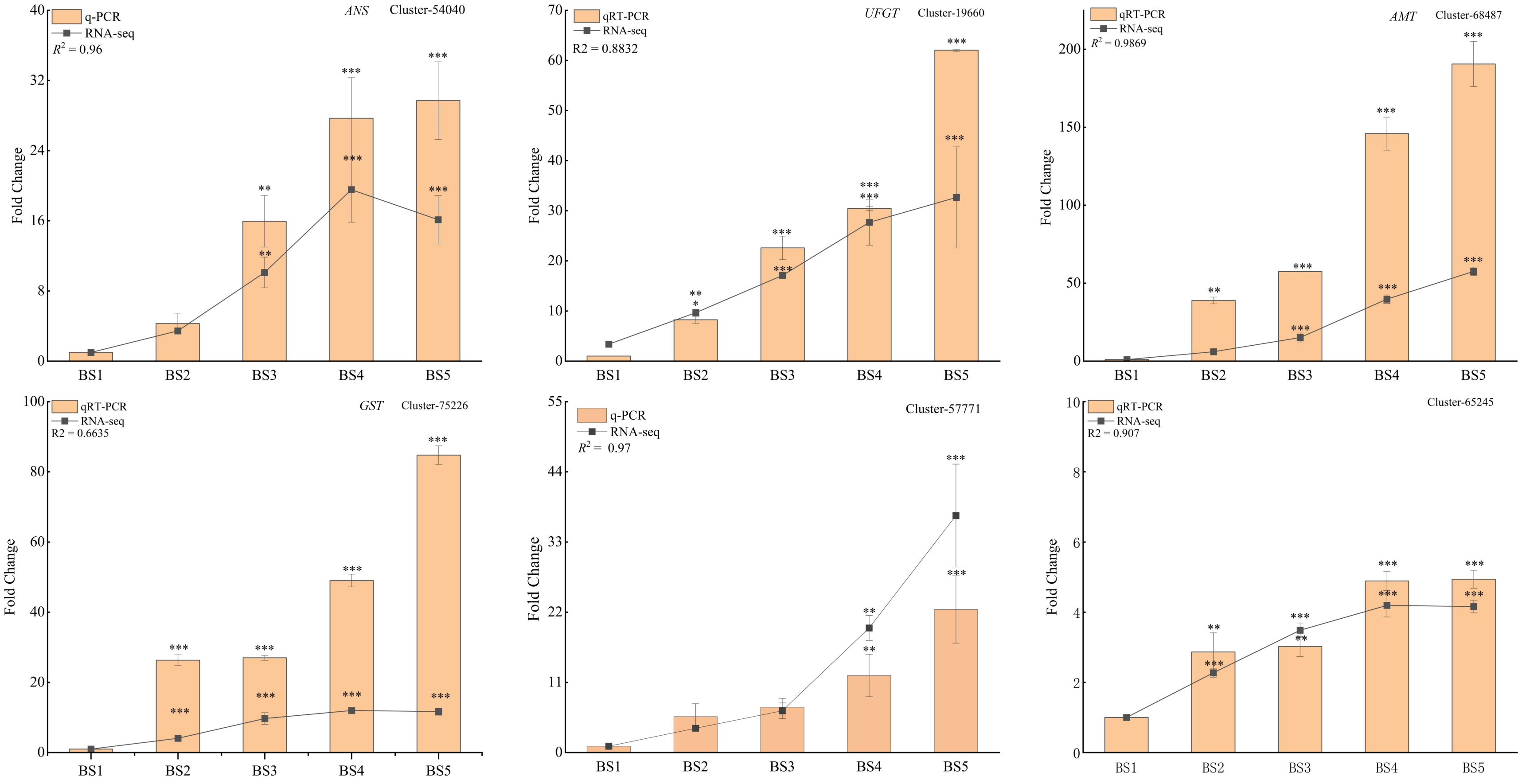
q-PCR
RNA-seq
R^2 = 0.96
ANS
Cluster-54040
Fold Change
BS1 BS2 BS3 BS4 BS5
qRT-PCR
RNA-seq
R2 = 0.8832
UFGT
Cluster-19660
Fold Change
BS1 BS2 BS3 BS4 BS5
qRT-PCR
RNA-seq
R^2 = 0.9869
AMT
Cluster-68487
Fold Change
BS1 BS2 BS3 BS4 BS5
qRT-PCR
RNA-seq
R2 = 0.6635
GST
Cluster-75226
Fold Change
BS1 BS2 BS3 BS4 BS5
q-PCR
RNA-seq
R^2 = 0.97
Cluster-57771
Fold Change
BS1 BS2 BS3 BS4 BS5
qRT-PCR
RNA-seq
R2 = 0.907
Cluster-65245
Fold Change
BS1 BS2 BS3 BS4 BS5

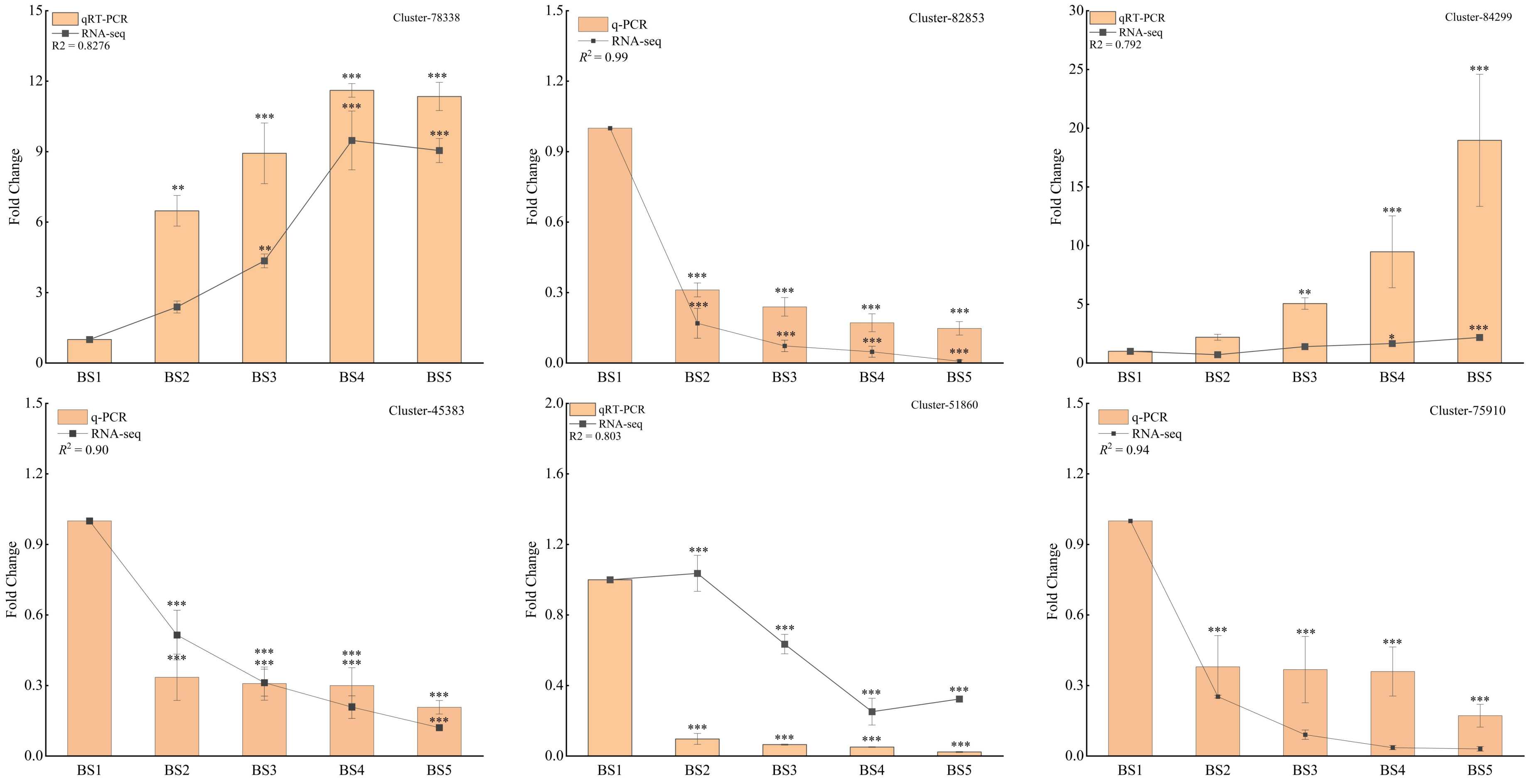

qRT-PCR
RNA-seq
R2 = 0.8276
Cluster-78338
Fold Change
BS1 BS2 BS3 BS4 BS5
q-PCR
RNA-seq
R^2 = 0.99
Cluster-82853
Fold Change
BS1 BS2 BS3 BS4 BS5
qRT-PCR
RNA-seq
R2 = 0.792
Cluster-84299
Fold Change
BS1 BS2 BS3 BS4 BS5
q-PCR
RNA-seq
R^2 = 0.90
Cluster-45383
Fold Change
BS1 BS2 BS3 BS4 BS5
qRT-PCR
RNA-seq
R2 = 0.803
Cluster-51860
Fold Change
BS1 BS2 BS3 BS4 BS5
q-PCR
RNA-seq
R^2 = 0.94
Cluster-75910
Fold Change
BS1 BS2 BS3 BS4 BS5

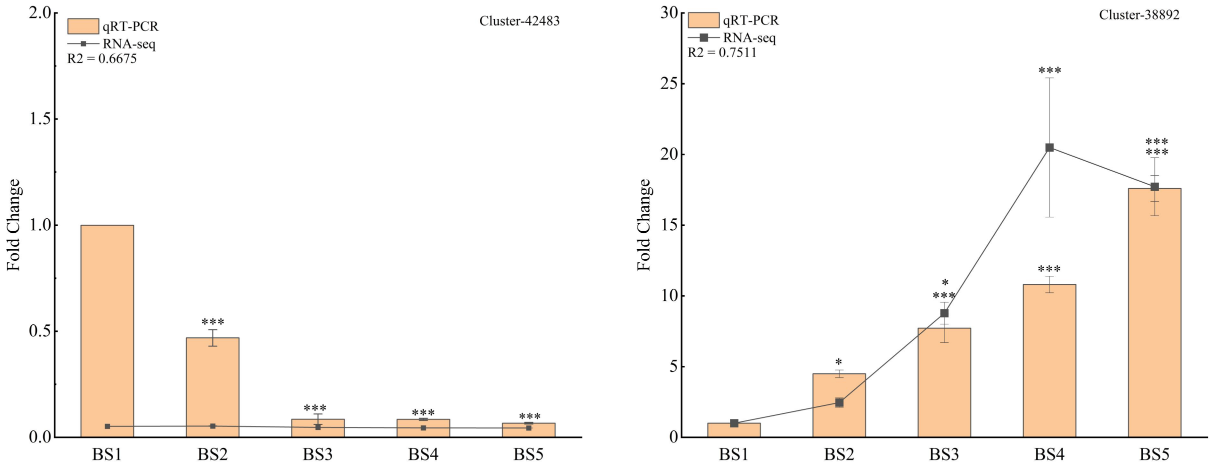

Figure S3. The gene expression assay of qRT-PCR. *, Significant difference ($p<0.05$); **, Highly significant difference ($p<0.01$); ***, Highly significant difference ($p<0.001$).
