# Supplementary material for: Integrated Metabolomic and Transcriptomic Analyses of Anthocyanin Synthesis During Fruit Development in Lycium ruthenicum Murr
Source: Biology (Basel). 2025 Nov 18;14(11):1614. doi: 10.3390/biology14111614 (PMC12650669; doi:10.3390/biology14111614)
Supplement: Supplementary file 1 [file biology-14-01614-s001.zip › Table S1.pdf]

Table S1 Primer List

| Gene ID | Sequence (5' to 3') | Product length | Tm(℃) |
| --- | --- | --- | --- |
| Cluster-68487 | TATGACAGGCAAAACCGCCC<br>ACATCGACAGGAATTGGGCT | 188 | 55 |
| Cluster-73437 | TCCTTTACGAGCACCCCAAG<br>GGCAAGTCTCAATGGCTCCT | 240 | 58 |
| Cluster-75226 | CATGGTACTCCAGCTCTTCGT<br>ACCTAAGGCTAGGGAGGTGG | 185 | 58 |
| Cluster-76458 | ACGACGTTGTGCTTTGTGTG<br>TCAGCTCCAGAAACGTGCTT | 132 | 58 |
| Cluster-38892 | AGCGGTTTTTCAAGGGGAGT<br>AGTTTGGTTGGAAGGACGCA | 243 | 58 |
| Cluster-65245 | CCACGAACCAGTAAACCCGA<br>GGATGATAGCTCCCTGGCAC | 149 | 58 |
| Cluster-78338 | CACCTATTCTCGCCAGTCCC<br>ACTGGCAACTTTGGAAGGCT | 152 | 58 |
| Cluster-42483 | AAATGTGCTGCTTGTGGCTG<br>AGATGGCGAAAACCGCCTAA | 117 | 58 |
| Cluster-51860 | AAGTCTCAAGTCGTCGTGGTC<br>CCTTTCGTTATGCCCAGCCA | 132 | 60 |
| Cluster-57771 | CAAGGAGGAAATGCAAAGAGGAGC<br>CTATTAGGGACCATCTGTTGCCAAG | 254 | 55 |
| Cluster-54040 | CTAAATGCCCCCAACCAGAACT<br>GTTACCCACTTCCCTTCATAGA | 124 | 58 |
| Cluster-50506 | GAACTACTGGTGAAGGGCTCGAATG<br>CTAAGCAGCAACACTGTGGAGGAC | 95 | 58 |
| Cluster-67748 | TCCCTTTTCCTTCCGAGTTCAT<br>TGTTTTGCCCTTCCACTGCTGC | 164 | 58 |
| Cluster-74326 | CAGTGGTGAACTCGGATAGCAGCA<br>GCTCCGCCATTACTGCTTTCTCTC | 117 | 58 |
| Cluster-67674 | AGAATGGGCACTGGCAGAAATGAT<br>ACTACACACGGCTCGTTTGATACC | 210 | 58 |
| Cluster-75910 | TCTACAAACGCCACAAGAACACTG<br>CCAAAACTCCTGTCTGTCTCCTTC | 142 | 58 |
| Cluster-82853 | CCAGATACAAAGTCATCATTCCCG<br>AACGATAAGGTATTCTTGGGAGGC | 154 | 58 |
| Cluster-45383 | ACAACCCATCAAAGCCAAAACC<br>AATGCGGAGTCACATGAGCTAAGAG | 163 | 58 |
| Cluster-19660 | CTGGAGAGAGTGAAAGACAAGGG<br>CCAAAATAGCCATCTTCATCCC | 251 | 58 |
| Cluster-84299 | GTCGGGGAGTTTTCTTCACAG<br>TTTTCAAGGAGAGGAATTGCTT | 149 | 58 |
| *LrH2B1* | AGTGCTTCCTGGTGAATTGG<br>TGGATAATACCTAGCCCTAGTTTCC | 163 | 58 |
