# Supplementary material for: Integrated Metabolomic and Transcriptomic Analyses of Anthocyanin Synthesis During Fruit Development in Lycium ruthenicum Murr
Source: Biology (Basel). 2025 Nov 18;14(11):1614. doi: 10.3390/biology14111614 (PMC12650669; doi:10.3390/biology14111614)
Supplement: Supplementary file 1 [file biology-14-01614-s001.zip › Table S2 .pdf]

Table S2. The relative content of the flavonoid in *L. ruthenicum* Fruit.

Index	Cla II	Relative Content (Log10)				
		BS1	BS2	BS3	BS4	BS5
mp001641	Anthocyanidin	6.67	6.68	6.33	7.42	7.90
pme3256	Anthocyanidin	6.39	7.69	7.55	8.51	8.65
pme2960	Chalcone	6.27	6.71	6.60	6.19	6.46
HJN055	Chalcone	6.17	6.30	6.39	6.94	7.08
mw2118	Chalcone	6.02	6.18	6.27	6.79	6.98
Zmpn004068	Chalcone	5.42	5.62	5.67	6.24	6.40
Lmn004797	Chalcone	5.09	5.16	5.10	5.41	5.24
Lmp004851	Chalcone	5.37	5.21	5.15	5.50	5.52
mw0355	Flavanol	4.67	5.22	5.13	4.90	5.07
mw1397	Flavanol	4.78	5.28	5.19	4.93	5.08
mw2220	Flavanol	4.27	4.34	4.29	5.32	5.50
mw0034	Flavanol	4.19	4.31	4.25	5.19	5.38
HJN087	Flavanone	4.36	5.09	5.65	6.36	6.53
Hmmn002588	Flavanone	3.79	3.65	3.59	5.04	5.33
mw1179	Flavanone	5.60	6.34	6.87	7.62	7.76
HJN086	Flavanone	5.40	5.76	5.63	5.86	5.87
HJN090	Flavanone	4.38	5.07	5.61	6.35	6.48
Hmmn002781	Flavanone	3.98	4.52	5.20	6.44	6.63
Zmhn001036	Flavanone	5.56	5.81	6.03	6.77	7.08
mw0046	Flavanone	4.90	5.45	6.19	7.64	7.81
pmb3023	Flavanone	5.39	5.79	5.80	5.88	5.79
pme1611	Flavanone	4.35	4.58	4.73	5.45	5.72

mw1066	Flavanone	4.62	5.32	6.01	7.45	7.64
HJAP135	Flavanone	4.93	5.15	5.05	5.90	5.67
pme1662	Flavanone	4.45	4.69	4.66	4.50	5.12
Hmmn002379	Flavanone	4.73	5.28	5.76	5.60	5.82
Lmtn002796	Flavanonol	5.42	5.78	5.83	5.98	5.89
mw0914	Flavanonol	6.09	6.48	6.35	5.93	6.15
HJN104	Flavanonol	4.74	4.92	4.91	5.86	6.70
Zmxp004503	Flavone	0.95	0.95	5.94	6.63	6.79
pmb2991	Flavone	3.60	3.59	3.83	4.85	5.08
Hmmp002324	Flavone	4.36	5.24	5.89	6.73	6.78
Hmmp002169	Flavone	5.38	5.72	5.93	6.36	6.48
Hmmp002322	Flavone	5.40	5.89	6.10	6.78	6.91
Hmmn003584	Flavone	4.50	5.03	5.56	5.64	5.61
pmb0716	Flavone	4.60	4.62	4.81	5.55	5.81
Lmtn002916	Flavone	4.52	4.72	4.60	5.01	5.02
Hmmp002073	Flavone	5.41	5.75	5.92	6.27	6.42
pmb3042	Flavone	5.25	5.48	5.87	6.96	7.26
pmb0620	Flavone	5.24	5.61	6.02	6.66	7.07
Lmhp003162	Flavone	4.79	5.21	5.37	6.22	6.46
pmb2987	Flavone	4.28	4.48	4.62	5.08	5.33
pma0249	Flavone	4.43	5.12	5.54	6.37	6.53
pmb0635	Flavone	4.29	4.68	4.99	5.76	5.90
Zmhp003186	Flavone	3.72	4.64	5.20	5.60	5.90
MWHY0122	Flavone	5.41	6.14	6.66	7.58	7.72
Hmgp002036	Flavone	6.19	6.23	6.67	7.04	7.14
Cwjp002289	Flavone	3.96	4.32	5.04	6.88	7.08

Hmmp002266	Flavone	4.68	4.98	4.98	4.77	4.87
Lmgp004959	Flavone	4.38	4.35	4.65	5.36	5.64
pmb3012	Flavone	4.95	4.91	5.11	5.89	6.07
pmb0672	Flavone	4.66	5.19	5.85	6.54	6.79
Lmyp004318	Flavonol	4.16	4.66	4.70	5.12	5.57
Hmln001836	Flavonol	6.04	6.30	6.51	7.22	7.12
Zmcn004206	Flavonol	5.54	5.74	5.53	6.05	6.07
Lmp004090	Flavonol	5.59	6.38	6.93	7.60	7.69
mw0032	Flavonol	4.79	0.95	5.63	6.87	6.93
Lmmp002334	Flavonol	4.41	4.21	4.81	6.37	6.54
Lmp003790	Flavonol	5.66	6.48	6.80	7.68	7.86
Hmcp001636	Flavonol	5.79	5.86	6.01	6.63	6.66
pmb0645	Flavonol	4.93	5.15	5.44	6.96	7.30
pmp001310	Flavonol	5.03	5.17	5.51	7.03	7.36
HJAP127	Flavonol	6.65	6.85	6.69	8.22	8.52
Hmcp001829	Flavonol	4.41	4.68	4.43	5.30	5.36
MWHY0162	Flavonol	4.95	5.11	5.32	6.97	7.31
HJAP128	Flavonol	5.49	6.17	6.72	7.49	7.66
Lmjp002906	Flavonol	6.31	6.35	6.57	7.04	7.23
mw0066	Flavonol	4.54	5.00	4.96	5.04	4.87
HJAP126	Flavonol	3.98	4.38	4.10	5.12	5.04
Hmmp002240	Flavonol	5.44	5.98	6.32	7.01	7.23
Hmcp001578	Flavonol	5.93	6.64	7.21	7.73	7.71
Zmcn003256	Flavonol	4.37	4.22	4.10	5.15	5.43
Zmgp002857	Flavonol	6.51	6.75	6.69	8.21	8.50
Lmdp002969	Flavonol	0.95	6.17	6.43	7.74	7.69

Hmcp002187	Flavonol	5.98	6.56	6.24	6.18	6.11
Zmcp002666	Flavonol	4.10	4.94	5.27	6.26	6.47
Hmcp001589	Flavonol	5.77	6.47	6.73	7.77	7.99
Lmp003252	Flavonol	5.21	5.69	5.28	6.20	6.55
Lmhp002374	Flavonol	4.36	5.18	6.01	7.87	8.09
Lmp004045	Flavonol	5.43	5.91	6.17	6.86	6.63
Lmbp002336	Flavonol	6.60	7.04	7.00	6.77	6.74
Hmcp002207	Flavonol	6.17	6.19	6.20	6.67	6.78
HJAP119	Flavonol	5.26	6.17	6.77	7.68	7.70
Lmjp003206	Flavonol	5.33	5.65	5.77	6.92	7.25
Lmmp002755	Flavonol	4.33	4.58	0.95	6.44	6.65
pmp000596	Flavonol	4.83	5.32	4.89	5.84	6.02
pmp001311	Flavonol	4.57	4.64	5.17	6.23	6.65
Lmp003729	Flavonol	4.47	5.41	5.17	6.18	6.08
Lmwp004293	Flavonol	3.81	3.80	4.87	5.70	5.84
HJAP123	Flavonol	3.75	3.94	0.95	6.22	6.82
Lmp003939	Flavonol	5.52	5.82	6.13	6.72	6.75
pmb0618	Flavonol	0.95	4.70	4.91	5.73	6.16
Zblp004717	Flavonol	6.81	7.12	7.15	6.98	6.81
Zmhp002640	Flavonol	3.85	4.23	4.38	5.96	5.88
Lmp003161	Flavonol	3.61	3.95	4.98	5.15	5.38
pmp001105	Flavonol	3.61	3.95	4.98	5.15	5.38
