# Supplementary material for: Integrated Metabolomic and Transcriptomic Analyses of Anthocyanin Synthesis During Fruit Development in Lycium ruthenicum Murr
Source: Biology (Basel). 2025 Nov 18;14(11):1614. doi: 10.3390/biology14111614 (PMC12650669; doi:10.3390/biology14111614)
Supplement: Supplementary file 1 [file biology-14-01614-s001.zip › Table S3 .pdf]

Table S3. The expression of transcription factors correlated with total anthocyanin.

TF-Family	Gene ID	FPKM					Correlation Coefficient	*p*-Value
		BS1	BS2	BS3	BS4	BS5		
zf-HD	Cluster-40940	72.82	104.88	52.92	33.46	34.08	-0.883	0.0470
	Cluster-42483	68.75	75.80	29.22	9.13	5.08	-0.966	0.0080
	Cluster-70239	14.30	9.15	5.97	2.32	2.00	-0.928	0.0230
WRKY	Cluster-84299	12.37	8.86	17.34	26.95	20.53	0.878	0.0500
Tify	Cluster-78338	3.88	9.27	16.89	36.81	35.14	0.983	0.0030
SBP	Cluster-51860	8.88	5.86	2.08	0.64	1.21	-0.886	0.0460
	Cluster-59293	37.46	33.56	21.23	9.61	7.20	-0.990	0.0010
OFP	Cluster-37929	4.12	5.15	4.87	20.94	22.44	0.947	0.0140
NF	Cluster-50033	3.87	3.25	2.76	0.42	0.07	-0.985	0.0020
	Cluster-45141	10.18	8.57	8.31	0.15	2.93	-0.912	0.0310
NAC	Cluster-53530	56.01	179.36	206.27	289.38	395.86	0.929	0.0220
	Cluster-58177	49.93	45.44	31.86	12.73	9.31	-0.997	0.0000
	Cluster-65112	13.96	16.67	8.29	2.51	5.03	-0.930	0.0220
	Cluster-65994	25.18	58.20	90.06	121.24	104.23	0.897	0.0390
	Cluster-66799	13.99	37.52	65.64	71.05	87.79	0.921	0.0260
MYB	Cluster-50220	0.15	0.00	0.34	0.42	0.59	0.933	0.0210
	Cluster-61965	10.80	10.56	10.38	29.93	39.43	0.934	0.0200
	Cluster-46349	0.79	2.11	2.76	19.83	29.51	0.948	0.0140
	Cluster-46489	7.28	10.84	8.18	3.24	2.99	-0.889	0.0440
	Cluster-57771	3.43	12.91	21.55	66.08	121.29	0.935	0.0200
MADS-MIKC	Cluster-38751	12.50	60.98	100.95	280.17	340.49	0.985	0.0020
LOB	Cluster-45094	2.15	6.79	17.91	249.37	413.13	0.936	0.0190

	Cluster-53828	23.45	159.95	264.21	483.17	732.94	0.966	0.0070
HSF	Cluster-11154	2.33	11.71	13.47	45.03	55.22	0.970	0.0060
	Cluster-57752	119.28	179.08	133.62	45.35	30.97	-0.905	0.0350
	Cluster-61892	17.70	69.18	107.40	142.75	163.43	0.948	0.0140
HB	Cluster-65245	34.67	78.84	120.91	145.45	144.32	0.909	0.0320
	Cluster-52223	2.79	3.72	6.75	41.00	92.92	0.892	0.0420
	Cluster-69422	55.37	64.54	85.44	193.82	319.50	0.933	0.0210
	Cluster-50530	8.73	14.59	11.35	31.18	54.18	0.891	0.0420
	Cluster-81545	27.16	19.19	57.02	124.78	106.03	0.963	0.0080
	Cluster-82754	42.36	50.79	47.20	24.20	18.55	-0.912	0.0310
	Cluster-73473	9.16	14.26	16.42	38.72	37.30	0.966	0.0070
GRAS	Cluster-34020	3.49	4.05	2.51	0.38	0.05	-0.988	0.0020
FAR1	Cluster-82260	0.02	0.26	0.00	1.12	2.04	0.889	0.0440
EIL	Cluster-66391	0.47	2.51	2.88	3.90	4.18	0.880	0.0490
DBB	Cluster-78339	6.48	17.97	18.67	34.11	41.72	0.954	0.0120
CPP	Cluster-67549	5.25	5.22	4.52	2.03	2.40	-0.970	0.0060
C3H	Cluster-80457	0.10	0.00	0.00	0.59	0.51	0.885	0.0460
C2H2	Cluster-66042	1.79	0.92	1.53	5.91	6.98	0.944	0.0160
C2C2-Dof	Cluster-38260	5.48	5.20	4.08	12.35	16.07	0.900	0.0380
bZIP	Cluster-46487	0.47	0.58	1.39	3.62	5.20	0.975	0.0050
	Cluster-55694	13.18	10.06	4.23	2.34	1.63	-0.931	0.0210
	Cluster-59123	6.73	8.79	19.51	105.43	161.99	0.952	0.0130
	Cluster-81368	44.32	45.91	28.13	11.16	14.34	-0.975	0.0050
bHLH	Cluster-45747	0.81	0.32	0.60	9.65	15.88	0.926	0.0240
	Cluster-76366	50.02	248.10	391.97	441.16	505.87	0.893	0.0420

	Cluster-76902	0.94	1.04	0.60	3.80	4.32	0.912	0.0310
	Cluster-82695	9.35	12.41	29.19	58.48	64.00	0.999	0.0000
B3	Cluster-46371	15.90	15.80	12.72	9.54	0.00	-0.905	0.0340
	Cluster-65027	1.86	1.99	1.30	0.48	0.97	-0.912	0.0310
	Cluster-66251	6.06	4.17	4.80	2.10	2.51	-0.879	0.0490
AP2/ERF	Cluster-46164	7.54	13.39	27.06	100.57	141.04	0.969	0.0060
	Cluster-50118	6.93	4.81	10.26	11.56	18.19	0.915	0.0290
	Cluster-55446	9.27	8.90	6.66	2.41	1.29	-0.998	0.0000
	Cluster-60203	40.76	76.57	74.98	162.42	184.49	0.961	0.0090
	Cluster-82855	59.57	56.36	31.17	8.51	3.62	-0.994	0.0010
	Cluster-38892	0.52	1.29	4.59	10.72	9.27	0.975	0.0049
